# Cases from Practice

**Published:** 1888-12

**Authors:** B. Simmons


### CASES FROM PRACTICE.

Case I.—During my residence in Cheltenham, England, I was called to attend two sisters, both suffering from measles. Up to the fifth day, the cases seemed of the ordinary character, but on the sixth, seventh, and eighth days, the patients becoming steadily worse, I was asked to re-visit them, when I found the following state present:

High fever, rapid pulse, great thirst, *with desire for wine or*

*beer ;* restlessness, complete sleeplessness caused by a feeling of intense *giddiness,* directly the patients attempted to *close their eyes ;* frequent convulsive cough, during which their heads were spasmodically jerked forward, the knees at the same time being jerked up toward the abdomen.

Their whole state gave me great anxiety, as I have seldom seen patients, after measles, so ill. Theridion $^{200}$ (Leip.) three globules in twelve teaspoonfuls of water, one teaspoonful every two hours, produced, within twenty-four hours, tranquillity, sleep, perspiration ; on the second day of the administration of the medicine, the interval between the doses was lengthened. Both patients made a rapid recovery. These cases were watched and nursed by a highly intelligent lady, who informed me that her doubts as to the efficacy of homœopathic medicines were at an end. The cough mentioned above is peculiar ; it disappeared under the influence of Theridion. The patients were total abstainers, but craved for the stimulants mentioned above, during the attack.

### Case II.—Did Homœopathy Fail?

G. A., aged fifty-two, called to wish me good-bye previous to her return to London. In the course of conversation, I learned from her that for a period of nine months, she had been suffering with neuralgia, of an intermittent character, and recurring daily. She stated that nearly the whole of this time she had been under the care of a homœopathic physician in London, who had informed her that in her case he had tried every known homœopathic remedy for neuralgia, including large doses of Quinine, but as they had all failed to help her, he advised change of air to Cheltenham. The air of Cheltenham did her no good, so that she had been obliged to admit to her friends that Homœopathy, in which she was a professed believer, had failed. I begged her to allow me to give her one prescription, and, obtaining her consent, noted down the following symptoms :

Aching in the right forehead, extending to the right eye, and forcing it to close ; pain in the occiput of a sudden, shooting character. The pain commences between nine and ten every morning, and lasts for about three hours, when it gradually disappears.

On page 348 of Jahr's *New Manual,* beginning line 17, I found my patient's symptoms almost verbatim (excepting the forenoon aggravation) under Calc. carb., and as the paroxysm was passing off, I placed upon her tongue a few globules of this remedy in a high potency (Fincke 107M) and gave placebo for fourteen days. At the end of that time, she wrote to me as follows : "I can never be sufficiently grateful to you for what you have done for me. On the day after I saw you I had a slight attack, since then I have been quite free from all pain." Three months later she reported herself well.

### Case III.—Zinc in Hysterical Retention of Urine.

E. G., aged thirty-five, was placed under my care, suffering from retention of urine. The patient was an hysterical woman, and complained of an immense number of aches and pains of an ever-changing character. For a period of nine years the urine had been drawn off by the catheter every morning ; the bowels were obstinately constipated. For some time I tried in vain to obtain the characteristics of her case—her pains moved rapidly from one part to another, and she always felt better in wet weather.

After nine months' unsuccessful treatment, she told me that on looking up she felt giddy and saw showers of gold descending, but hoped I would not laugh at her, as every medical man had done to whom she had mentioned the symptom. On page 704, Lippe's *Materia Medica*, line 21, under Zinc, I found the following : "When lifting up the eyes he sees luminous flakes." I decided to give her a long course of this remedy, prescribing three globules of Zincum$^{200}$ (Jenichen), in nine teaspoonfuls of water, one teaspoonful three times a day. At the end of the third month of this treatment the bladder acted spontaneously, the constipation disappeared, and the general health of the patient was wonderfully restored. She remained under my supervision for several years—the use of the catheter was never again required.

### Case IV.—A Berberis Case.

G. A., aged fifty-two, consulted me for what he called rheumatic pains in the legs. He told me that he was an old homœopath, and he was disappointed to find that the remedies he had taken had had no beneficial influence over his disease. He felt that he was losing his walking power, being unable to proceed further than about a hundred yards at a time. After he had walked a short distance he was compelled to stop from a feeling of *intense weariness, heaviness, lameness and stiffness of the legs, which felt sore, as if bruised.*

He expressed great anxiety about his condition, as he had great responsibilities and feared he should be compelled to give up his profession. On page 278, Jahr's *New Manual*, under

Berberis, I found an exact description of my patient's case (lines 1, 2, 3, lower extremities). I gave the patient one dose of Berberis$^{70m}$ (Fincke). Improvement set in on the fourth day, and in a fortnight he was well. For the last twelve months this gentleman's walking power has been completely restored. Soon after I commenced the practice of Homœopathy a gentleman consulted me with precisely the same symptoms as were present in the above case. I prescribed many remedies, but did not give the right one. I failed to cure, a misfortune which I am persuaded would never have occurred had a suitable dose of Berberis been admininistered.

B. SIMMONS, M. D.

